# Quantum interference between transverse spatial waveguide modes

**DOI:** 10.1038/ncomms14010

**Published:** 2017-01-20

**Authors:** Aseema Mohanty, Mian Zhang, Avik Dutt, Sven Ramelow, Paulo Nussenzveig, Michal Lipson

**Affiliations:** 1 School of Electrical and Computer Engineering, Cornell University, Ithaca, New York 14853, USA; 2 Department of Electrical Engineering, Columbia University, New York, New York 10027, USA; 3 School of Engineering and Applied Sciences, Harvard University, Cambridge, Massachusetts 02138, USA; 4 School of Applied and Engineering Physics, Cornell University, Ithaca, New York 14853, USA; 5 Institute for Physics, Humboldt-University Berlin, 12489 Berlin, Germany; 6 Instituto de Fisica, Universidade de São Paulo, P.O. Box 66318, São Paulo 05315-970, Brazil; 7 Kavli Institute at Cornell for Nanoscale Science, Cornell University, Ithaca, New York 14853, USA

## Abstract

Integrated quantum optics has the potential to markedly reduce the footprint and resource requirements of quantum information processing systems, but its practical implementation demands broader utilization of the available degrees of freedom within the optical field. To date, integrated photonic quantum systems have primarily relied on path encoding. However, in the classical regime, the transverse spatial modes of a multi-mode waveguide have been easily manipulated using the waveguide geometry to densely encode information. Here, we demonstrate quantum interference between the transverse spatial modes within a single multi-mode waveguide using quantum circuit-building blocks. This work shows that spatial modes can be controlled to an unprecedented level and have the potential to enable practical and robust quantum information processing.

## Abstract (web summary)

Practical implementations of quantum photonic circuits consist primarily of large waveguide networks to path-encode information. Here, Mohanty *et al*. demonstrate quantum interference between transverse spatial modes in a single silicon nitride waveguide, enabling robust quantum information processing.

Integrated quantum optics has drastically reduced the size of table-top optical experiments to the chip scale, allowing for demonstrations of large-scale quantum information processing and quantum simulation^1^,^2^,^3^,^4^,^5^,^6^,^7^. However, despite these advances, practical implementations of quantum photonic circuits remain limited because they consist of large networks of waveguide interferometers that path encode information, but do not easily scale. Increasing the dimensionality of current quantum systems using higher degrees of freedom, such as transverse spatial field distribution, polarization, time and frequency to encode more information per carrier will enable scalability by simplifying quantum computational architectures^8^, increasing security and noise tolerance in quantum communication channels^9^,^10^, and simulating richer quantum phenomena^11^. These degrees of freedom have previously been explored in free-space and fibre quantum systems to encode qudits and implement higher-dimensional entanglement^12^,^13^,^14^,^15^,^16^.

Currently, integrated quantum photonic circuits are primarily limited to path-encoding information, but the use of a higher-dimensional Hilbert space within each path will increase the information capacity and security of quantum systems. Higher dimensionality allows one to encode more information per photon, relieving resource requirements on photon generation and detection^9^,^10^. Consequently, this leads to more efficient logic gates and noise-resilient communications, making quantum systems more scalable and practical^8^,^17^. In integrated schemes, a few demonstrations have been developed for polarization^18^ and time^19^. In free-space optics, orbital angular momentum and Hermite–Gaussian modes have both been used to encode information within a higher-dimensional space as qudits (*d*-level logic units)^12^,^13^,^14^,^15^,^16^. The higher-order waveguide modes in a multi-mode interferometer have been used to passively mix single-mode inputs for quantum interference, and transfer polarization and path-encoded states^20^,^21^. However, the spatial modes have never been controlled individually to encode quantum information to date^22^,^23^. The transverse spatial degree of freedom is an untapped resource that can be manipulated using simple photonic structures and does not require exotic material properties.

In the classical regime, the orthogonal spatial modes of an integrated waveguide have been shown to markedly scale data transmission rates^24^,^25^,^26^,^27^,^28^. A waveguide can support many co-propagating modes, which can be used as parallel channels within a single waveguide. Progress in the field has overcome the challenge of achieving controlled coupling while avoiding unwanted coupling between different modes, for example, in bends and tapers^29^,^30^. Mode conversion based on waveguide structuring has significant potential in the quantum regime^31^,^32^,^33^.

Here, we demonstrate a scalable platform for photonic quantum information processing using waveguide quantum circuit-building blocks based on the transverse spatial mode degree of freedom: spatial mode multiplexers and spatial mode beamsplitters. A multi-mode waveguide is inherently a densely packed system of spatial and polarization modes that can be coupled by perturbations to the waveguide. We design a multi-mode waveguide consisting of three spatial modes (per polarization) and a nanoscale grating beamsplitter to show tunable quantum interference between pairs of photons in different transverse spatial modes. We also cascade these structures and demonstrate NOON state interferometry within a multi-mode waveguide. We show that interference between different transverse spatial waveguide modes and active tuning can be achieved with high visibility using this platform. These devices have potential to perform transformations on more modes and be integrated with existing architectures, providing a scalable path to higher-dimensional Hilbert spaces and entanglement.

## Results

### Hong–Ou–Mandel interference using spatial waveguide modes

**Figure 1:**
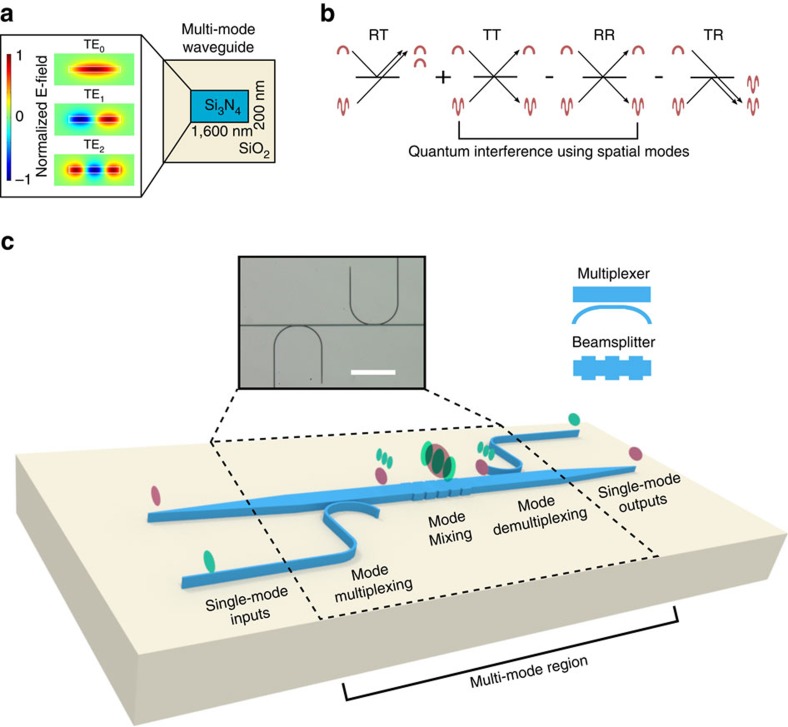
Quantum interference using a spatial mode beamsplitter. (**a**) Simulation of transverse spatial modes in a multi-mode waveguide. (**b**) Schematic shows interference between two indistinguishable photons incident on the two input ports (or modes) of a beamsplitter, where the two input ports are the two spatial modes of a waveguide (TE0 and TE2). The four cases of probability amplitudes, in which TE0 and TE2 are reflected (R) or transmitted (T) are added or subtracted based on the unitary transformation of a beamsplitter. The arrows indicate whether the photons remain in the same mode or convert to the other mode. The destructive interference of the two cases that result in coincidences (RR and TT) leads to the characteristic HOM interference. (**c**) Schematic showing chip implementation of spatial mode multiplexing (asymmetric directional coupler) and spatial mode beamsplitter (nanoscale grating). The colours indicate the mode order within the multi-mode region of the device (red is TE0, green is TE2). The colour also shows the path that transfers single-mode inputs and outputs to the different spatial modes within the multi-mode waveguide. Wavelength (808 nm) and polarization (TE) are identical within each path. The inset shows a microscope image of the device. Scale bar is 160 μm.

To show the potential utility of the integrated transverse spatial degree of freedom for scalable quantum information processing, we demonstrate Hong–Ou–Mandel (HOM) interference between two different quasi-transverse electric (TE) waveguide modes (TE0 and TE2). HOM interference is a useful proof of principle because it is the basis of many other quantum operations, such as higher-dimensional entanglement, teleportation, quantum logic gates and boson-sampling^1^,^2^,^3^,^4^,^15^,^16^,^34^. In the original HOM experiment, a path beamsplitter is used to combine two originally orthogonal paths of two single photons, making them indistinguishable. The probability amplitudes of the two cases that contribute to detection of the two photons in coincidence destructively interfere owing to the bosonic nature of photons, if the two paths are indistinguishable^35^. As an example, we consider a silicon nitride multi-mode waveguide with a sub-micron cross section containing six modes: three spatial modes per polarization (Fig. 1a). In our experiment, we replace the path beamsplitter with a spatial mode beamsplitter and replace the two paths with two spatial modes within a multi-mode waveguide (Fig. 1b). The spatial mode beamsplitter couples two different spatial modes, resulting in a superposition of the two spatial modes. Mode coupling leads to interference within the waveguide between the cases, in which both photons remain in their original modes or both couple to opposite modes (cases RR and TT in Fig. 1b). Visibility of the interference in coincidences is a measure of the equal splitting in the beamsplitter and indistinguishability of the two paths in every degree of freedom, including transverse spatial mode.

The key building blocks required to demonstrate HOM interference are a spatial mode multiplexer for generating the different spatial modes and a spatial mode beamsplitter for interfering the spatial modes, which both rely on selective mode coupling by phase matching in our design. The spatial mode multiplexer allows us to generate orthogonal spatial modes within the multi-mode waveguide without cross talk between the modes, which would reduce the interference visibility. We couple pairs of photons from a spontaneous parametric down conversion (SPDC) source into single-mode silicon nitride waveguides that couple into a multi-mode waveguide (Methods; Fig. 1c). Finally, the photons are sent to the spatial mode beamsplitter where they are equally split between the two modes, coupled into single-mode output waveguides, and the fundamental mode fields are detected as coincidences. We use a silicon nitride platform because the high-core-cladding (Si_3_N_4_/SiO_2_) index contrast allows one to strongly vary the propagation constants of different spatial modes by varying the waveguide dimensions, which is essential for selective mode coupling. The silicon nitride platform is attractive for integrated quantum information processing because its transparency window spans the visible to mid-infrared wavelength range and has been used to demonstrate non-classical light sources^36^,^37^.

### Selective mode coupling by phase matching

**Figure 2:**
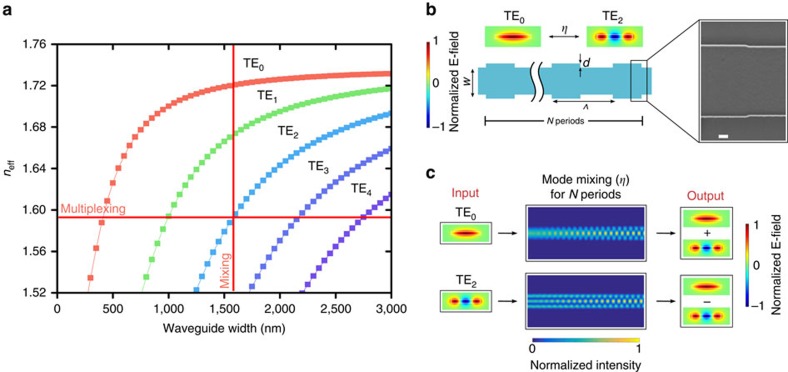
Design of spatial mode beamsplitter. (**a**) Si_3_N_4_ dispersion for a multi-mode waveguide with 190 nm height. Horizontal red line shows phase matching for waveguides with different widths for spatial mode multiplexing. Vertical red line shows phase matching between modes in a single waveguide for the spatial mode beamsplitter. (**b**) Symmetric grating structure for coupling the TE0 and TE2 modes. The period is defined by the difference in effective index between the modes in a particular waveguide. The period (*Λ*) is 6.675 μm and the grating depth, *d*, is 24 nm. The width, *w*, is 1,600 nm and the height is 190 nm. Inset: SEM of fabricated grating structure. Scale bar, 200 nm. (**c**) Simulation of mode conversion in a 50:50 splitter for *η*=0.5, where *N*=20 periods and all other dimensions are the same as in **b**.

To demonstrate the spatial mode multiplexer, we use an asymmetric directional coupler to selectively couple the fundamental mode in a single-mode waveguide to a specific higher-order mode in an adjacent multi-mode waveguide. The asymmetric directional coupler uses two different waveguide widths to phase match light propagating in different modes within adjacent waveguides, allowing for efficient coupling^24^,^25^,^26^,^27^. In Fig. 2a, the horizontal red line indicates where the effective indices of different higher-order modes in waveguides of different widths match. For example, to excite the TE2 mode in the multi-mode waveguide using the TE0 mode in a single-mode waveguide with 420 nm width, we choose the multi-mode waveguide width of 1.6 μm (Methods).

To demonstrate the spatial mode beamsplitter, we use a nanoscale grating structure to selectively couple different higher-order spatial modes within a multi-mode waveguide. The period of the grating structure provides a momentum change that accounts for the phase mismatch between the two different spatial modes^38^. In Fig. 2a, the vertical red line indicates the phase mismatch (Δ*n*_eff_) between modes within a single waveguide, *Λ*=*λ*/Δ*n*_eff_ where *Λ* is the period of the grating, *λ* is the wavelength and *n*_eff_ is the effective index of the mode. For example, to couple TE0 and TE2, Δ*n*_eff_=0.12 and *Λ*=6.675 μm. We define the splitting ratio, *η*, as the probability of coupling to the same mode, and 1−*η* as the probability of coupling to the opposite mode. This splitting ratio can be tuned from 0 to 100% if the two modes are perfectly phase matched. This splitting ratio (*η*) depends on the coupling coefficient (*κ*) determined by the overlap of the two modes within the perturbed region (grating depth, *d*) and the length of the coupling interaction (or the number of periods, *N*) as follows: *η*=sin^2^(*κN*) (Fig. 2b; Supplementary Note 1). We use finite element method and EigenMode expansion to determine the phase matching and splitting ratios. Figure 2c shows a simulation of a 50:50 coupler between TE0 and TE2. Beamsplitters with tunable splitting ratio are crucial building blocks for photonic quantum simulation circuits^3^,^4^,^39^ and for reconfigurable quantum circuits for quantum metrology and processing^2^.

### HOM interference visibility measurements

**Figure 3:**
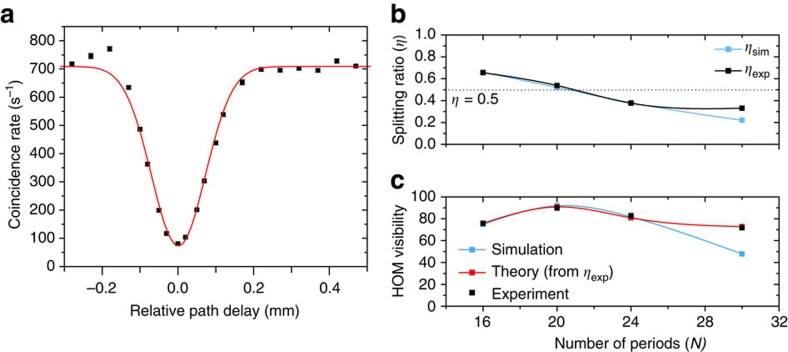
Hong–Ou–Mandel interference between TE0 and TE2. (**a**) We show the coincidence rate (accidentals subtracted) of the two output arms as we delay one input arm. The red line is a Gaussian fit to the experimental data. The HOM visibility is 90±0.8%. The error bars indicate the standard error of measurement and are not visible because they are smaller than the data points. (**b**) Comparison between experiment and simulation of the splitting ratio as the number of periods (*N*) is varied. Error bars on experimental data are smaller than data points. (**c**) Corresponding HOM visibility as the number of periods (*N*) is varied.

**Figure 4:**
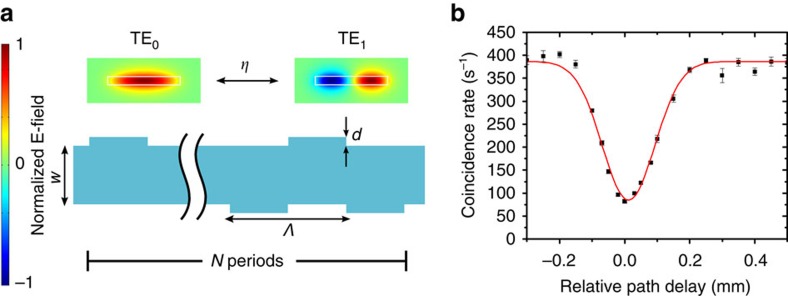
Hong–Ou–Mandel interference between TE0 and TE1. (**a**) Schematic of asymmetric grating to couple even to odd modes. The period (*Λ*) is 8 μm, the number of periods (*N*) is 8 and the grating depth, *d*, is 24 nm. The width, *w*, is 1,010 nm and the height is 190 nm. This corresponds to a splitting ratio *η*=0.64. (**b**) We show the coincidence rate (accidentals subtracted) of the two output arms as we delay one input arm. The red line is a Gaussian fit to the experimental data. The HOM visibility is 78±0.3%. The error bars indicate standard error of measurement.

We observe a high HOM visibility of 90±0.8% between photons sent through the TE0 and TE2 mode channels. In Fig. 3a, coincidences with accidentals subtracted are plotted against relative path length difference between the two input arms, and the best Gaussian fit is indicated by the red curve. The device with splitting ratio near 1/2 (where *N*=20), yields the highest visibility of 90±0.8% with a coherence length of 168±10 μm, which we estimate from the width of the coincidence dip. This device is primarily limited by the source visibility, which we measure to be 92% (Methods) due to spectral mismatch of the two arms. With an ideal source, this device could have a high visibility of 99% with a measured splitting ratio of *η*_exp_=0.55. A discussion on the effect of loss and cross talk on the visibility can be found in Supplementary Note 2. In Fig. 3b, we show measured splitting ratios near 1/3, 1/2 and 2/3 for devices with different numbers of coupling periods, which agree well with simulations. These ratios have been of particular importance in path-encoded implementations of controlled-NOT gates in quantum photonic circuits^1^. Note that the device with the longest-coupling interaction does not produce as much splitting as predicted by simulations, which is most likely due to residual phase mismatch. As expected, we show that the experimental HOM visibilities depend on the splitting ratios measured and agree well with their theoretical visibilities from the measured splitting ratios (Fig. 3c). To show that this method easily extends to other modes of different parities, we also demonstrate a visibility of 78±0.3% between TE0 and TE1. We use an asymmetric grating for a structure that is limited to 78% by its splitting ratio (*η*_exp_=0.64; Fig. 4).

**Figure 5:**
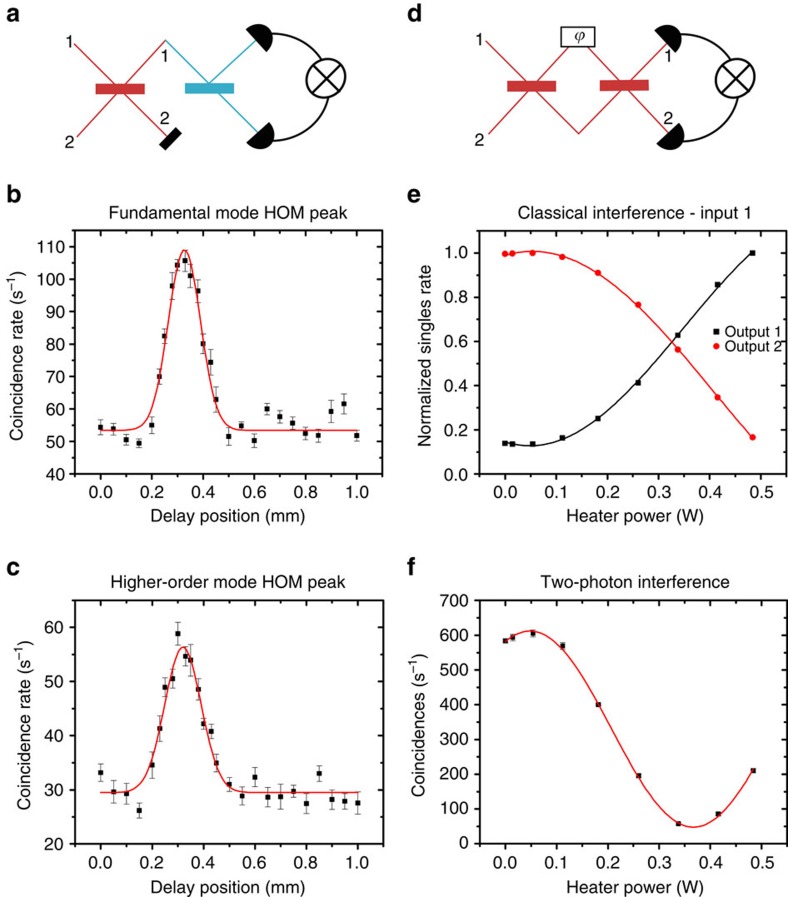
Hong–Ou–Mandel peak and NOON interference. (**a**) Schematic shows HOM peak experiment arrangement. Red indicates on-chip spatial mode beamsplitter, and blue indicates fibre beamsplitter. Pairs of photons are simultaneously coupled into the fundamental mode (input port 1) and higher-order mode (input port 2) of the on-chip spatial mode beamsplitter. To determine the fundamental mode HOM peak, the photons from the fundamental mode (output port 1) are sent to a fibre beamsplitter, and coincidences are measured using detectors and coincidence logic. The same is done for the higher-order HOM peak using output port 2. (**b**) Coincidence rate of the fundamental mode output arm after the fibre beamsplitter. There is a peak in coincidences due to HOM bunching. (**c**) Coincidence rate of the higher-order mode output arm after the fibre beamsplitter. (**d**) Schematic shows NOON interference experiment arrangement. Red indicates the on-chip spatial mode beamsplitter. Pairs of photons are simultaneously coupled into the fundamental mode (input port 1) and higher-order mode (input port 2) of the on-chip spatial mode beamsplitter. The two photons are sent to the spatial mode phase shifter based on an integrated microheater that applies a phase shift (*φ*) between the fundamental and higher-order modes. Finally, coincidences are measured using detectors and coincidence logic. (**e**) Classical Mach–Zehnder interference is shown as a function of heater power that applies the phase shift. (**f**) NOON interference is shown as a function of heater power. The period of the quantum interference is half that of the classical interference. The error bars indicate standard error of measurement for all plots.

To further confirm the observed HOM effect, we measure photon coalescence enhancement at the individual output arms of the HOM interferometer^15^,^16^. We expect a doubling of the probability of case TR (TE0 transmitted, TE2 reflected) and RT (TE0 reflected, TE2 transmitted) in the HOM experiment in comparison to the experiment with distinguishable photons (Fig. 1b). We use a fibre beamsplitter at the individual output arms of the spatial mode beamsplitter (*η*=0.55) and measure coincidences. Figure 5a–c shows a peak in coincidences for both the fundamental and higher-order mode output port with a visibility of 2±0.02 that matches well with theory. The width of the multi-mode HOM peak is 166±10 μm, and the width of the single-mode output is 147±10 μm. This effect has been used as a basis for quantum cloning experiments^15^.

### NOON interference visibility measurements

Finally, to show these structures can be cascaded and actively tuned, we fabricate a Mach–Zehnder structure to create a NOON state interferometer based on our spatial mode beamsplitter^40^. The HOM interferometer and phase shifter produces the NOON state described by: 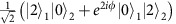 where *φ* is the phase between the two modes of the interferometer, and the subscripts 1 and 2 refer to the different modes. NOON states are more generally written as 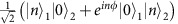 and provide increased phase sensitivity, *φ*, by 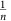 for quantum metrology over the standard quantum limit of 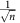 (ref. 40). In our experiment, the Mach–Zehnder structure consists of two gratings separated by a phase shifter, a length of waveguide and heater (Methods; Supplementary Fig. 1). Within the phase shifter, the waveguide is tapered out to 10 μm width that gives a larger differential in phase shift between the fundamental and higher-order modes as the heater is tuned. In Fig. 5d–f, we show measurements of the classical interference by inputting a single arm of the SPDC source into the device and measuring the single counts of both output arms, which show the classical Mach–Zehnder fringe as expected. This specific device (*η*_exp_=0.66) has a classical visibility of 82±8% with a period of about 1.3±0.082 W, which corresponds to the power of the heater. The relatively high powers required to achieve a differential phase shift between the higher-order modes requires further optimization. Simulations and extended discussion on this point are included in Supplementary Note 3 and Supplementary Figs 2 and 3. We then measure the two-photon interference, or NOON state interference, by measuring coincidences when both arms of the SPDC source are input into the device with no path delay. We observe a visibility of 86±1% with a period of 0.64±0.005 W, about half of the classical interference. In addition to the increased phase sensitivity, this demonstrates the active tunability of this device, which could be useful in state preparation for quantum simulators^3^,^4^,^39^.

## Discussion

In this work, we show a step perturbation that has a frequency response that includes additional higher-order frequencies. Because we have limited our multi-mode waveguides to the three lowest-ordered modes, these higher-frequency components do not pose problems. When dealing with a larger number of modes that require couplings given by multiple spatial frequency components, a sinusoidal perturbation would ensure less cross talk between the modes. These devices for two-mode couplings could be cascaded to create arbitrary transformations between modes. This initial demonstration between two modes can be extended to make arbitrary *n*-dimensional unitary matrix transformations on a set of modes, which is essential for quantum information processing and simulation^41^. We include an example of designing a three-mode splitter and an extended discussion on the footprint and scalability of this platform in Supplementary Note 4 and Supplementary Figs 4 and 5. Assuming 5 nm fabrication tolerance on dimensions, we can realistically expect to multiplex at least 15 modes within a silicon nitride waveguide^42^. This number of quantum modes corresponds to a Hilbert space with a dimensionality of 15^2^=225 for a two-waveguide system. Higher-index materials will increase the number of modes that can be multiplexed. These designs could also be made more compact by using multiplexed gratings, in which the perturbation has multiple spatial frequency components and strengths to design arbitrary transformations of the modes^28^,^31^,^32^,^33^. This same analysis can be extended to the quasi-transverse magnetic (TM) polarized spatial modes, and adiabatic tapers and asymmetric gratings can be used to convert between TE and TM polarized modes^21^,^43^. Combining spatial mode encoding with polarization and path encoding can further increase the Hilbert space of the integrated waveguide platform.

We show that these structures are tunable and can be cascaded, while preserving high-visibility quantum interference, which will be key to building larger networks. Multi-mode waveguides can be used with other degrees of freedom to encode information within a high-dimensional Hilbert space using only linear passive devices within a small footprint. These miniaturized systems with high information density could eventually be interfaced with spatial mode multiplexing in fibre and free-space systems for quantum information processing and could be specifically useful in quantum repeaters, memories, and simulators.

## Methods

### Device design and characterization

We use silicon nitride waveguides to implement the spatial mode multiplexer and spatial mode beamsplitter. The device has inverse tapers (∼170 nm) to mode match to 2 μm spot size of tapered fibres. The single-mode waveguides are 190 nm tall and 420 nm wide. The single-mode waveguide is tapered adiabatically (100 μm long taper) to the multi-mode waveguide, which is 190 nm tall and 1,600 nm wide. We use COMSOL and FIMMWAVE software packages to simulate the mode profiles and coupling. The asymmetric directional coupler has a coupling length of 18 μm between the single-mode and multi-mode waveguide. The perturbation needed to couple the modes in the multi-mode waveguide is quite small, ∼ 24 nm, in order to remain within the weak coupling regime (Fig. 2b), but large enough to yield reasonable device lengths. For gratings with 24 nm depth, the coupling coefficient is (*κ*=0.041) per period. The simulation shows ∼50:50 coupling for *N*=20, corresponding to a device length of ∼ 133 μm for our specific geometry. We estimate the loss in the device excluding coupling losses to be 0.2 dB. To characterize the on-chip beamsplitters and fibre beamsplitters, the classical splitting ratios (*η*) were measured using an 808 nm diode laser source.

### Device fabrication

We deposit 190 nm of low-pressure chemical vapour silicon nitride on a silicon wafer with 4 μm of thermal oxide. Then, we pattern with electron beam lithography and etch the waveguides. We finally clad the devices with 300 nm of high-temperature oxide and 2 μm of plasma-enhanced chemical vapour-deposited silicon dioxide. For the cascaded device with the integrated phase shifter, we fabricate the heater (50 nm Ni) and contact pads (200 nm Al) using a metal lift-off process.

### Experimental set-up for interference

**Figure 6:**
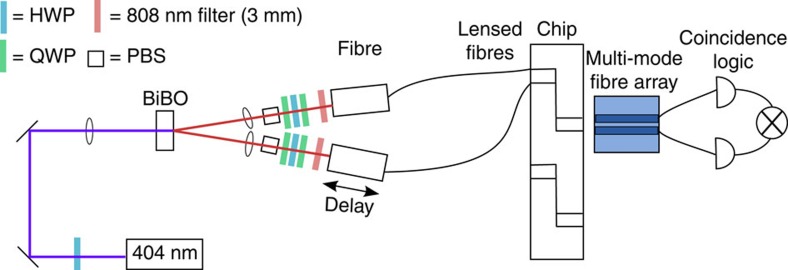
Spontaneous parametric down conversion (SPDC) source and coincidence counting set-up. The photon pair source is coupled to the chip using lensed fibres and coupled to the coincidence counting setup using a fibre array. The polarization is filtered using a series of a half-wave plate (HWP), quarter-wave plate (QWP) and polarizing beamsplitter (PBS).

To observe the HOM interference, we couple photon pairs into the spatial mode beamsplitter on the chip and measure coincidences. We produce degenerate 808 nm photon pairs using SPDC (Type I) by pumping a bismuth borate crystal with a 404 nm diode laser (Fig. 6). We use polarizing beamsplitters, waveplates, and bandpass filters (3 nm) to couple indistinguishable photon pairs into the chip using a lensed fibre. The output of the beamsplitter is collected using a multi-mode fibre array and sent to the single-photon counting modules (SPCM-AQRH) and coincidence logic (Roithner TTM8000). We manually adjust the delay by translating one of the fibre couplers at the source with a micrometre screw and use a coincidence window of 2 ns to minimize accidental coincidences. The SPDC source HOM visibility was characterized using a single-mode fibre beamsplitter, and we measured a visibility of 92±1.9% and coherence width of 194±10 μm. We attribute this reduced visibility primarily to spectral differences between the two arms. The HOM peak experiment uses a multi-mode fibre beamsplitter and detected coincidences with the same coincidence window. Finally, to test the Mach–Zehnder structure, we apply a voltage on the heater using a Keithley sourcemeter to produce the phase shift between the modes (Supplementary Note 3).

### Data availability

The data that support the findings of this study are available within the article and the Supplementary Information file.

## Additional information

**How to cite this article:** Mohanty, A. *et al*. Quantum interference between transverse spatial waveguide modes. *Nat. Commun.* **8,** 14010 doi: 10.1038/ncomms14010 (2017).

**Publisher's note**: Springer Nature remains neutral with regard to jurisdictional claims in published maps and institutional affiliations.

## Supplementary Material

Supplementary Information Supplementary Figures, Supplementary Notes and Supplementary References

Peer Review File
