# Supplementary material for: Quantum interference between transverse spatial waveguide modes
Source: Nat Commun. 2017 Jan 20;8:14010. doi: 10.1038/ncomms14010 (PMC5263888; doi:10.1038/ncomms14010)
Supplement: Supplementary Information — Supplementary Figures, Supplementary Notes and Supplementary References [file ncomms14010-s1.pdf]

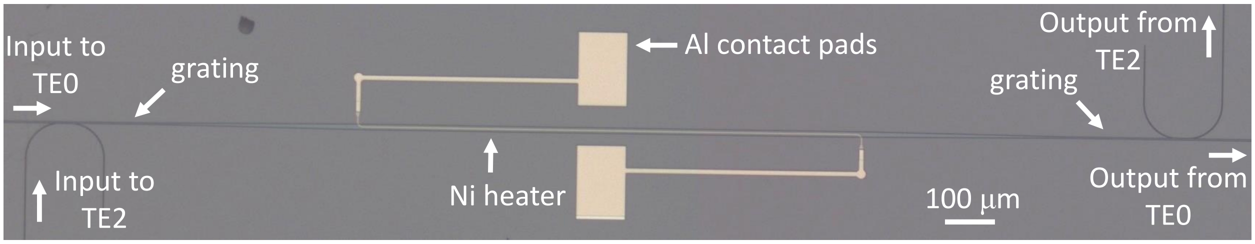

Supplementary Figure 1: NOON State Device Configuration. This microscope image of the NOON state device shows how the Mach-Zehnder interferometer is formed. First, the two inputs are sent to the mode multiplexer which sends one input to the fundamental mode (TE0) and the second input to the higher-order mode (TE2). Next, the two modes are mixed at the spatial mode beamsplitter (or nanoscale grating). The two modes are then sent to the phase shifter (Ni heater). Finally, the two modes are mixed at a second spatial mode beamsplitter and sent to single-mode outputs through the mode demultiplexer. Scale bar is 100 μm.

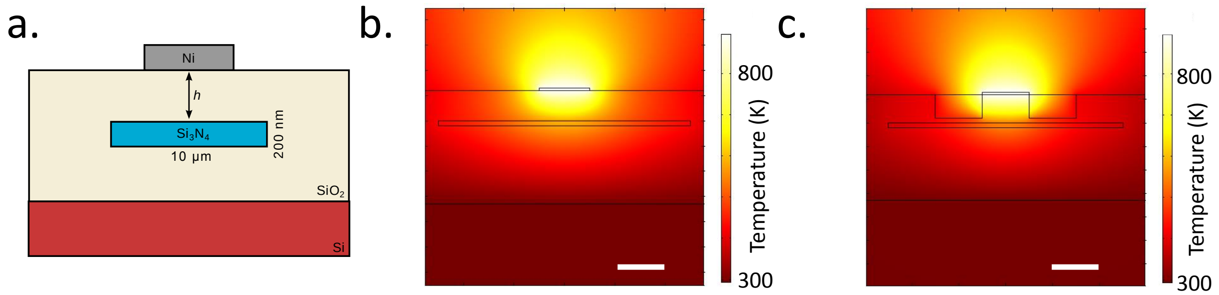

Supplementary Figure 2: Thermo-Optic Phase Shifter Cross-Section. a) Schematic shows the cross-section of the thermo-optic phase shifter which consists of a heater placed above the waveguide with a heater distance of h. The thermo-optic effect changes the refractive index of materials as a function of temperature, which leads to an accumulation of additional phase. In this experiment, the phase shifter consists of a waveguide region that is increased to 10 μm with a heater above the waveguide with SiO_2 cladding, where h is 2.3 μm. The heater is made of Ni, and the contact pads are made of Al. b) FEM simulation of heat distribution from the heater where h is 1.2 μm. Simulation is shown for a heater distance of 1.2 μm which doubles the heater efficiency. Scale bar is 2 μm. c) FEM heat distribution from the heater with a heater distance of 1.2 μm and air trenches to provide stronger gradient in temperature across the waveguide. The air trenches are indicated here by the rectangles adjacent to the heater. This strong thermal gradient across the waveguide leads to a greater relative phase shift between the fundamental and higher-order modes of the waveguide. Scale bar is 2 μm.

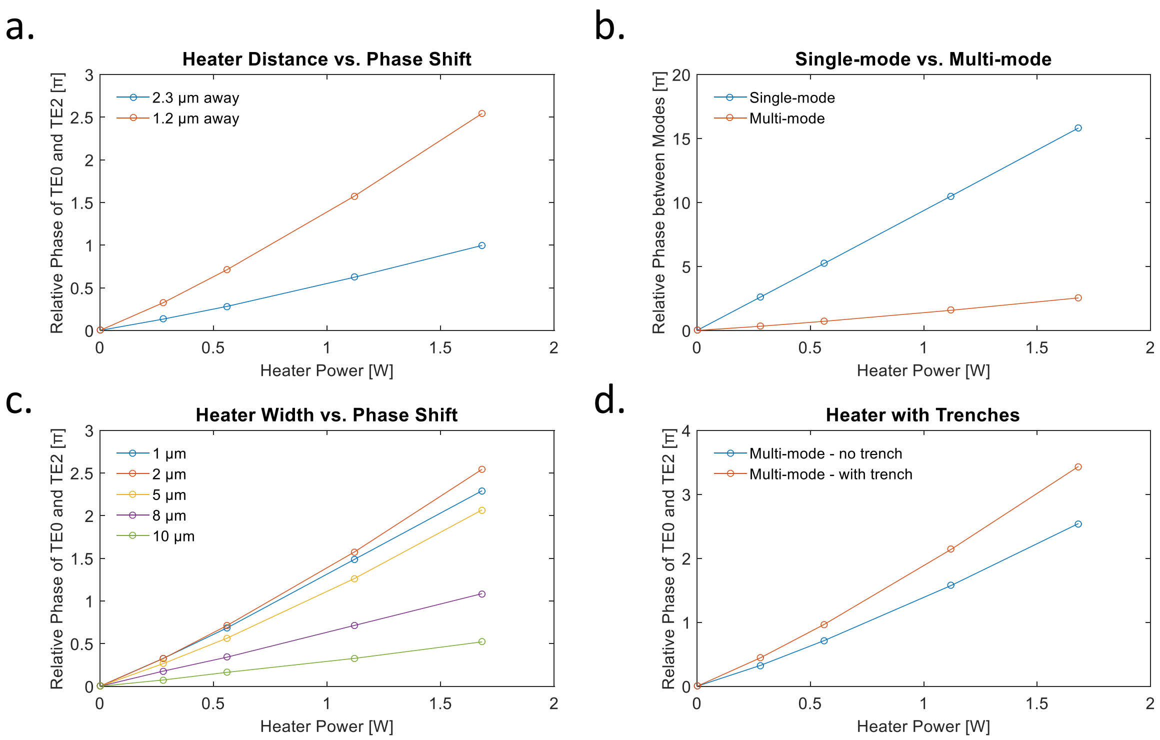

Supplementary Figure 3: Numerical Simulations of Thermo-Optic Phase Shifting. a) Shows the calculated relative phase shift between TE0 and TE2 for two different heater distances as a function of heater power. The heater power for a π phase shift is reduced by more than half by decreasing the heater distance from 2.3 µm to 1.2 µm. b) Shows the calculated relative phase difference between two single-mode paths and two spatial modes within a multi-mode waveguide as a function of heater power. The heater power required for a π phase shift is about 7 times larger for the multi-mode phase shifter than a single-mode phase shifter because the heat within the multi-mode waveguide interacts with all the spatial modes to some extent. c) Shows the relative phase shift between TE0 and TE2 for different heater widths as a function of heater power. In this experiment, we address the phase shifter efficiency issue by using a localized heater with a width of 2 µm. The plot shows this is the optimal heater width for a 10 µm wide waveguide. The heater power required for a π phase shift is reduced by 80% when the width is decreased from 10 µm to 2 µm. d) Shows the relative phase shift between TE0 and TE2 for a structure with and without trenches as a function of heater power. Air trenches surrounding the heater provide a strong thermal gradient across the waveguide which increases the relative phase between the spatial modes. The power required for a π phase shift is reduced by 25% by adding the air trenches.

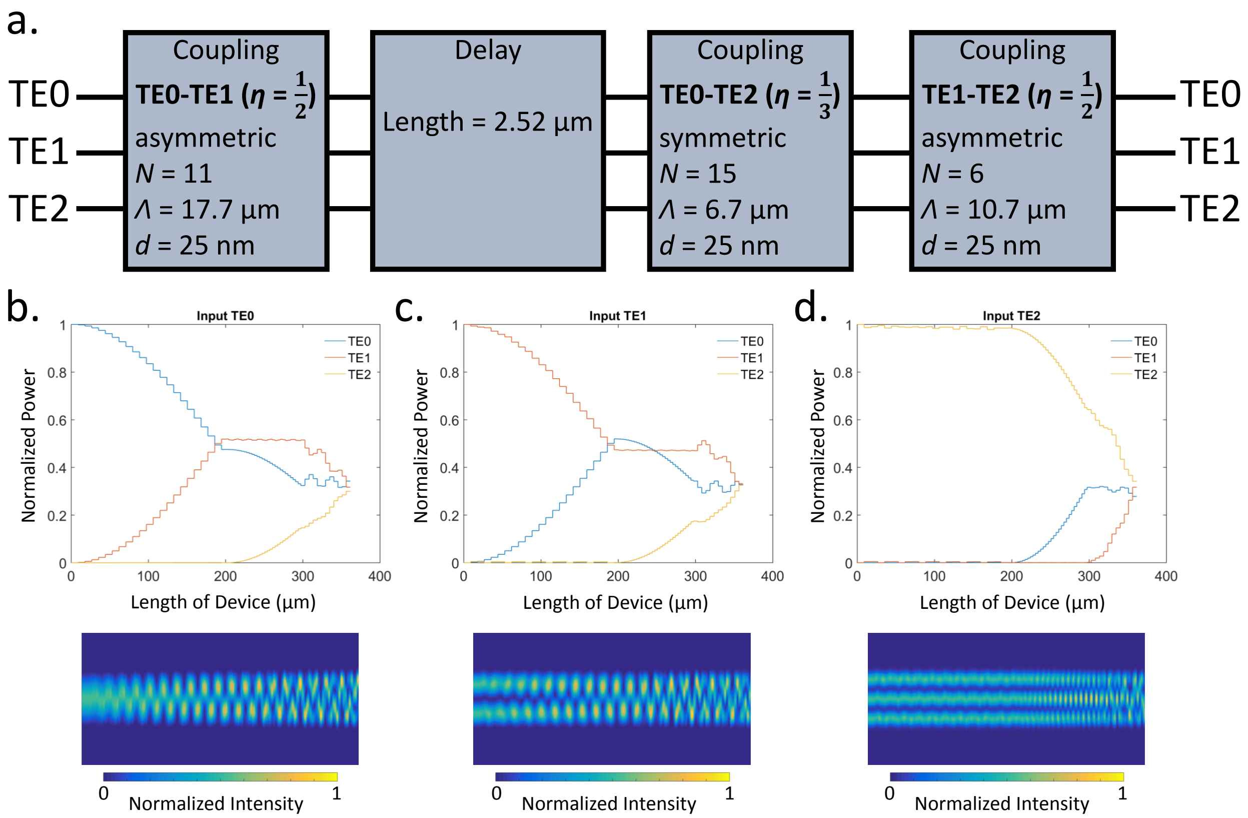

Supplementary Figure 4: Three Mode Splitter Example. a) Design of a three mode splitter using cascaded two mode couplings. The parameters are given for asymmetric or symmetric gratings as defined in the main text (see Fig. 2 and Fig. 4 from main text). b) Numerical simulation of the normalized mode powers when TE0 is input into device from part a. Below is the total normalized intensity distribution along the structure. c)Numerical simulation of the normalized mode powers when TE1 is input into device from part a. Below is the total normalized intensity distribution along the structure. d)Numerical simulation of the normalized mode powers when TE2 is input into device from part a. Below is the total normalized intensity distribution along the structure.

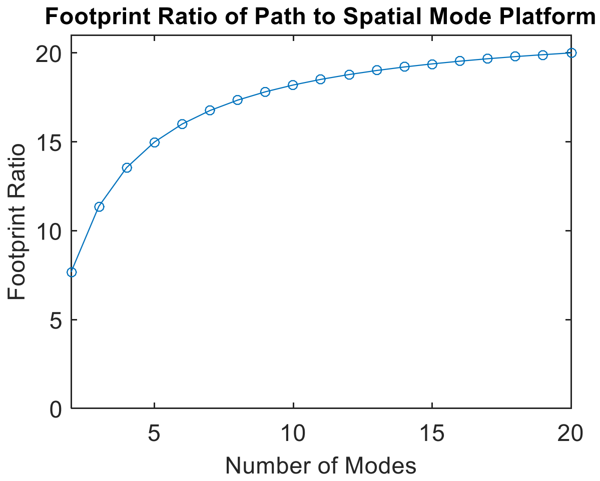

Supplementary Figure 5: Footprint comparison of path to spatial mode encoding using 2x2 transformations. We compare the ratio of system footprints for the same number of 2x2 operations as a function of the number of modes. We assume silicon nitride waveguides with 200 nm height and a path-encoded beamsplitter with 55 µm × 18 µm.

Supplementary Note 1: Coupled-Mode Analysis for Periodic Perturbation Mode Coupling

Coupled-mode theory for weakly coupled modes has been derived in many textbooks. Here we adapt the derivation from Haus.[1] Coupled-mode theory relates the complex amplitudes of modes a_1 and a_2 through a set of differential equations, where β_1 and β_2 are the uncoupled modes propagation constants and κ_{12} and κ_{21} are the coupling coefficients between the two modes.

$$\frac{\mathrm{d}a_1}{\mathrm{d}z} = -j\beta_1 a_1 + \kappa_{12} a_2 \tag{1}$$

$$\frac{\mathrm{d}a_2}{\mathrm{d}z} = -j\beta_2 a_2 + \kappa_{21} a_1 \tag{2}$$

The solution to these equations assuming that the waves $a_1(0)$ and $a_2(0)$ are launched at z=0 is the following:

$$a_1(z) = [a_1(0)(\cos\beta_0 z + j\frac{\beta_2 - \beta_1}{\beta_0}\sin\beta_0 z) + \frac{\kappa_{12}}{\beta_0}a_2(0)\sin\beta_0 z]e^{-j[\frac{\beta_1+\beta_2}{2}]z} \tag{3}$$

$$a_2(z) = [\frac{\kappa_{21}}{\beta_0}a_1(0)\sin\beta_0 z + a_2(0)(\cos\beta_0 z + j\frac{\beta_1 - \beta_2}{\beta_0}\sin\beta_0 z)]e^{-j[\frac{\beta_1+\beta_2}{2}]z} \tag{4}$$

where

$$\beta_0 = \sqrt{(\frac{\beta_1 - \beta_2}{2})^2 + |\kappa_{12}|^2} \tag{5}$$

If, initially, $a_1(0) = 1$ and $a_2(0) = 0$, the power coupled from a_1 to a_2 is:

$$|a_2(z)|^2 = |a_1(0)|^2 \left|\frac{\kappa_{21}}{\beta_0}\right|^2 \sin^2\beta_0 z \tag{6}$$

Here we define the splitting ratio as the following:

$$\eta = \frac{|a_2(z)|^2}{|a_1(0)|^2} = \left|\frac{\kappa_{21}}{\beta_0}\right|^2 \sin^2\beta_0 z \tag{7}$$

For perfect phase matching where $\beta_1 = \beta_2$, this becomes:

$$\eta = \sin^2|\kappa_{21}|z \tag{8}$$

For the periodic perturbation, the coupling occurs at each perturbation, so we can convert $|\kappa_{21}|$ (coupling per length) to κ (coupling per period) to get:

$$\eta = \sin^2\kappa N \tag{9}$$

where N is the number of periods.

Supplementary Note 2: Visibility Degradation due to Loss and Cross-talk

The primary sources of visibility degradation, aside from the non-ideal spontaneous parametric downconversion source, are the asymmetric loss of the spatial mode beamsplitter and the cross-talk of the mode multiplexer.

The cross-talk originates from the residual coupling away from the optimal phase matching point. In our case, we measure a cross-talk of less than -29 dB from the mode multiplexing. This leads to a non-ideal splitting ratio which degrades the visibility by approximately 0.002%. We also show that these devices can be cascaded and maintain high visibility through tapering structures consisting of a larger number of modes. For the quantum NOON state, we measure a visibility of $86 \pm 1\%$. The splitting ratio (η) for this device is 0.66 which results in an ideal visibility of $2\sqrt{\eta(1-\eta)}$ which is about 94.7%. If we take into account our source visibility of 92%, the estimated visibility is 87% which is within the error of our measurement. There is no significant degradation of the quantum visibility due to additional spatial modes in the NOON state interferometer, and this degradation comes solely from the deviation of the splitting ratio from 0.5.

The loss that contributes to the degradation of the visibility comes from an asymmetry in the loss between the supermodes (or coupled modes) within the beamsplitter. Here, we follow the derivation from Atwater for a lossy beamsplitter.[2] The theoretical maximum visibility is:

$$V = 1 - 2\cos^2 \Delta\theta \tag{10}$$

where $\Delta\theta$ comes from the phase difference between the reflected and transmitted modes. As derived in Atwater, the phase difference is:

$$\Delta\theta = 2\tan^{-1}(e^{\frac{\pi \Delta n_{\mathrm{i}}}{2\Delta n_{\mathrm{r}}}}) \tag{11}$$

where Δn_{i} is the difference of the imaginary effective index of the supermodes, and Δn_{r} is the difference of the real effective index of the supermodes. We estimate $\Delta n_{\mathrm{r}} = 1.6 \times 10^{-3}$ which we calculate from our coupling coefficient. As a worst case scenario, we attribute all of the 0.2 dB loss to the difference in the imaginary effective index to get $\Delta n_{\mathrm{i}} = 2.19 \times 10^{-5}$. We estimate this degrades the visibility by less than 0.1%. This is an overestimate of the difference in loss between the supermodes because the supermodes should have similar interactions with the sidewalls of the waveguide. The sidewall roughness from etching is the primary source of scattering loss. This can be further improved by using a waveguide width that is larger which minimizes the sidewall interaction further.

Supplementary Note 3: NOON State Device Design

The NOON state device consists of a Mach-Zehnder configuration of the spatial mode beamsplitters and phase shifter (see Supplementary Fig. 1 and Supplementary Fig. 2a). In the following section, we discuss methods to optimize the efficiency of the phase shifter.

The power required for this phase shift could be reduced by bringing the heater closer to the waveguide. In Supplementary Fig. 2b, we show COMSOL numerical simulations that calculate the temperature distribution and uses the thermo-optic coefficient to recalculate the effective index of the optical mode, which we use to calculate the relative phase shift of different modes. For example, according to Supplementary Fig. 3a if the distance is changed to 1.2 µm away, which still does not affect the optical mode, the heater power for a π phase shift is reduced by more than half.

If we compare the relative phase shift between the TE0 and TE2 modes in a multi-mode waveguide and the relative phase shift between two single-mode waveguides used in path-encoding using the same path length and heater distance, the power required for a π phase shift in the multi-mode waveguide is about 7 times larger (see Supplementary Fig. 3b). This occurs because the modes are spatially co-located, and the thermo-optic refractive index change affects both modes to some degree. However, one should note that this is the first proof-of-principle demonstration using a simple design technique. The efficiency of the differential phase shift induced by the heaters can be improved using the techniques we discuss here. In this work, we address this issue by using a localized microheater. As shown in Supplementary Fig. 3c, we used a heater width of 2 µm which shows the most efficient phase shifter. The heater power is reduced by 80% by using a heater that is 2 µm rather than 10 µm because the heat is localized and heats the TE0 mode more than TE2.

To reduce this power even further, trenches can be used to provide a stronger thermal gradient across the waveguide and apply a greater phase shift between the different modes. In Supplementary Fig. 3d, we show that the trenches reduce the power by 25% for an overall power reduction of about 90% from the results of this experiment.

Our analysis here has been limited to thermo-optic phase tuning. However, other electro-optic effects could be more localized. Further research in this area should include intermediate structures that utilize the inherently different coupling coefficients to apply larger differential phase shifts between the spatial modes.

Supplementary Note 4: Extended Discussion of Scalability

Arbitrary NxN mode transformations can be made using cascaded 2x2 beamsplitters and phase shifters.[3] In the mode multiplexing platform proposed, gratings can be used as 2x2 beamsplitters and short lengths of waveguide can be used as passive phase shifters. The gratings can be designed using selective phase matching, as outlined in the main text. No extra components are necessary because the waveguide modes are co-propagating and do not need additional routing.

Here, we show a design and numerical simulation for a three mode splitter based on 2x2 splitters using a single multi-mode waveguide with the same dimensions as the device in the main text (height = 200 nm, width = 1.6 µm) that contains three modes (TE0, TE1, and TE2). The design consists of three grating sections that couple two modes with different ratios depending on the grating depth, d, and period, Λ (see Supplementary Fig. 4a). Here we use two asymmetric gratings to couple even to odd modes (TE0-TE1 and TE1-TE2) and a symmetric grating to couple two even modes (TE0-TE2); the final transfer matrix is theoretically as follows where $b = e^{(\pm j2\pi/3)}$:

$$U = \frac{1}{\sqrt{3}} \begin{bmatrix} 1 & 1 & 1 \\ 1 & b & b^* \\ 1 & b^* & b \end{bmatrix} \tag{12}$$

The network of 2x2 beamsplitters offers the most flexibility and control of the NxN transformation. Using this approach, we can compare the estimated footprint of the path-encoded platform and the mode-encoded platform for a larger number of modes. Assuming silicon nitride waveguides with oxide cladding, the footprint of a path-encoded beamsplitter based on a 2x2 multi-mode interferometer (MMI) structure with S-bends is approximately 1000 µm^2. We estimate the area of the mode-encoded beamsplitter using FEM simulations for the width scaling and an estimate of the coupling coefficient. For five modes, the path-encoded structure is about 15 times larger than the mode-encoded structure for the same number of operations. We estimate the ratio of the footprint of a path-encoded system to a mode-encoded system with increasing number of modes (see Supplementary Fig. 5). This ratio saturates because we have only included horizontal higher-order modes which increases the width of the waveguide.

However, a compact MMI splitter for 4x4 can be made.[4] In this case, the footprint is about 4 times larger than the mode-encoded version in our work (using 2x2 transformations), and this ratio slowly increases with the number of modes. This occurs because as the width of the MMI is increased to incorporate more modes, the length to form the constructive images increases as well. To our knowledge for $N > 4$, it becomes difficult to experimentally realize an MMI with a balanced output and low insertion loss due to phase errors in the multi-mode section and the approximation necessary for the reimaging principle. Ideally, these MMI structures are simple structures, but they are primarily limited to symmetric splitting transformations. It has been suggested in literature to use gratings similar to those proposed in our work within the multi-mode section of the MMI to control the modes and produce variable splitting ratios.[5] Hence, the techniques we suggest here can be leveraged to improve path-encoded MMI designs as well.

There are many routes to scaling our mode-multiplexing platform even further by simply changing the geometry of these structures. Currently, in the mode-multiplexing community, the number of modes is increased by slightly increasing the width of the waveguide. However, by increasing the height of the waveguide, we can increase the number of modes while keeping the footprint constant. For example, if the 200 nm height of the silicon nitride waveguide in this paper is increased to 600 nm, twice as many modes are included, which can approximately halve the footprint.

The true potential of this platform can be realized by taking advantage of the fact that since these modes are co-propagating within the same waveguide, any mode can be accessed at any point without extra bends or waveguide crossings that induce loss. Multiplexed gratings can be used to simultaneously perform multiple couplings between different modes within the multi-mode waveguides. This can dramatically reduce the mode-encoded footprint to that of a single stage. These footprints can be as small as 40 µm^2, making them about 100 times smaller for the same number of modes of a path-encoded system.[6–8] The challenge will be to make compact devices such as these that are still highly tunable.

Supplementary References

[1] Haus, H., *Waves and fields in optoelectronics,* 1st edition (Prentice Hall, 1984).

[2] Fakonas, J. S., Lee, H. Kelaita, Y. A. and Atwater, H. A. Two-plasmon quantum interference. *Nat. Photon.* **8,** 317–320 (2014).

[3] Reck, M., Zeilinger, A., Bernstein, H. J. and Bertani, P. Experimental realization of any discrete unitary operator. *Phys. Rev. Lett.* **73,** 58–61 (1994).

[4] Bachmann, M., Besse, P. A. and Melchior, H. General self-imaging properties in NxN multimode interference couplers including phase relations. *Applied Optics* **33,** 3905–3911 (1994).

[5] Tseng, S.-Y., Fuentes-Hernandez, C., Owens, D. and Kippelen, B. Variable splitting ratio 2x2 MMI couplers using multimode waveguide holograms. *Opt. Express* **15,** 9015–9021 (2007).

[6] Ohana, D. and Levy, U. Mode conversion based on dielectric metamaterial in silicon. *Opt. Express* **22,** 27617–27631 (2014).

[7] Lu, J. and Vučković, J. Objective-first design of high-efficiency, small-footprint couplers between arbitrary nanophotonic waveguide modes. *Opt. Express* **20,** 7221–7236 (2012).

[8] Frellsen, L. F., Ding, Y., Sigmund, O., Frandsen, L. H. Topology optimized mode conversion in a photonic crystal waveguide fabricated in silicon-on-insulator material. *Opt. Express* **22,** 8525–8532 (2014).
