# Supplementary material for: Quantum interference between transverse spatial waveguide modes
Source: Nat Commun. 2017 Jan 20;8:14010. doi: 10.1038/ncomms14010 (PMC5263888; doi:10.1038/ncomms14010)
Supplement: Peer Review File [file ncomms14010-s2.pdf]

**Editorial Note:** this manuscript has been previously reviewed at another journal that is not operating a transparent peer review scheme. This document only contains reviewer comments and rebuttal letters for versions considered at Nature Communications.

Reviewers' comments:

Reviewer #1 (Remarks to the Author):

There remains to be revised again.
Chap.2
It'd be better to show the photo of the device.
=> If the space allows, it'd be better to add not in the in the supplementary info but in Fig.1 of the main text.

Chap.4
The visibility of NOON state interferometer degrades to 82%, compared with 90% of the single device. Based upon this degradation the authors are advised to discuss the device cascadability with respect to the number of guided modes.
=>
To the comment above, the authors didn't addressed this issue. I don't see the discussion in the supplementary info (section2) as is stated in Response to Reviewers.

Reviewer #2 (Remarks to the Author):

1. I am happy with the passive mode conversion schemes pointed out by the authors, though as far as I know there is no experimental demonstration of such an arbitrary mode converter yet. In principle, a careful design and engineering of the device probably can achieve this challenging task. The costs probably are the fabrication of the nano-structures and computational complexity of design and optimization.

2. The discussion of active mode-MZI here is focusing on how to exploit high TO-materials and engineer the structure to reduce the power consumption of the mode-MZI. However, using these technical improvements, I am suspicious that the power consumption for a standard MZI can be reduced as well. Thus, the authors should compare this mode MZI with the normal MZI within the same scope.

As the authors state in the main text that"… phase shift between the fundamental and higher order modes as the heater is tuned…". When increasing the temperature, the refractive indices of both modes increase, partially cancelling the phase difference between them. This could be the reason that relative high power consumption is necessary to induce the phase difference between different modes. Also, this mode MZI would be difficult to implement in high dimensional case.

3. The motivation of this work is to move to higher dimensional quantum systems by the use of many orthogonal waveguide modes, though this work is a demonstration of two modes case, it will be necessary to include a detailed discussion on how to scale it up using this technology.

4. The authors have compared the directional path-BS with the new mode-BS in term of device footprint. It will be even more convincing to compare this mode-BS directly with the multimode interferometer path-BS, which are both naturally utilizing the multimodes of waveguide.

5. I do agree that path-encode and mode-encode would/should not be exclusively competing with each other. Indeed, the use of mode-encoding here could add more functionality for chip-scale

quantum photonics. Together with the recent demonstration of polarization, mode, path encoding conversion in L.-T. Feng, et. al. Nat Commun 7, 11985 (2016) and other related works, it is exhibiting the promise future of potential quantum information applications.

--

Work in a similar vein of that recently been published in Nature Communications, where Feng et. al. reported an "On-chip coherent conversion of photonic quantum entanglement between different degrees of freedom", and used them to demonstrate the two-photon two-mode HOM and entanglement transfer and manipulation.

To achieve the mode-BS, Feng et. al. used a polarization-mode conversion scheme, while here this work exploited a new grating structure as the mode-BS. Feng et al acknowledge in their paper that "while this report was being written, quantum interference between transverse waveguide modes was realized" – referencing this work. It would appear the paper under review has taken much longer to get to press than the Feng paper, although it was perhaps performed at a very similar time. So, although the work is somewhat similar in goal, this is an important topic and I would suggest that it should be published in Nat Communications, providing a nice contrast to the work of Feng..

Ideally I would have liked for the authors to realize higher mode demonstration to verify their claims of scalability and show its advantage of using their mode-BS and mode-multiplexer for useful high dimensional quantum information systems.

Reviewer #4 (Remarks to the Author):

The manuscript details the demonstration of optical information processing on a chip making use of spatial modes and has already been through a round of review. The results are well presented and clear. Technical questions raised in the previous round of refereeing have been satisfactorily addressed by authors. Since the remaining questions seem to be over impact and novelty I'll focus on this aspect.

Pretty much every conceivable degree of freedom of the optical field has been exploited for photonic quantum information processing in free space optics. The most common are path and polarisation, but also angular momentum, spatial mode, frequency, temporal time-binning, and various combinations have been used. Some degrees of freedom are currently easier to manipulate with high precision than others but the driving force to explore others is the need to drastically scale up optical QIP and any advantage in doing so may prove useful. It is also clear that, other than for quantum communication, integrated optical devices are the path forward. To date there is comparatively little exploration of alternative degrees of freedom of the light field in integrated devices so the manuscript is immediately novel in this sense. The fact that the interference experiments demonstrated (HOM dip and two-photon NOON state) are not novel and not of record breaking fidelity is not the important point, the important point is that the transverse spatial modes can be manipulated with high fidelity and relatively easily. This is a first-in-class demonstration of such techniques.

More than this, the achieved fidelities are respectable. They are certainly not the highest, especially in comparison with free space, but then those technologies have benefitted from decades of technical development. The state of the art is barely more than a handful of photons and to do something truely useful may require hundreds if not thousands of photonic qubits so it is much too early to rule out any particular approach. The importance of the demonstrated experiment is that it shows the same lithography technology used in creating path encoded

devices can also give access to spatial mode encoding. If this is to be the eventual preferred approach to photonic QIP can't be said but it brings much needed diversification of approaches. Comparisons with other specific experiments that where raised by the previous referees have been addressed satisfactorily.

My recommendation is to accept the manuscript for publication.

*Reviewers' comments:*

*Reviewer #1 (Remarks to the Author):*

*There remains to be revised again.*
*Chap.2*
*It'd be better to show the photo of the device.*
*=> If the space allows, it'd be better to add not in the in the supplementary info but in Fig.1 of the main text.*

We add an inset microscope image of the device. However, the detail of the grating will not be visible, and will only be visible in the close-up SEM in figure 2.

*Chap.4*
*The visibility of NOON state interferometer degrades to 82%, compared with 90% of the single device. Based upon this degradation the authors are advised to discuss the device cascadability with respect to the number of guided modes.*
*=>*
*To the comment above, the authors didn't addressed this issue. I don't see the discussion in the supplementary info (section2) as is stated in Response to Reviewers.*

As described previously in the response to the reviewers, the quantum visibility of the device degrades from 90% to 86% as stated in lines 159-160 in the main text. The 82% which the reviewer has mentioned corresponds to the classical visibility which had a larger margin of error. We included a discussion in the supplementary section 2 to explain this. The following is the statement in the supplementary: *For the quantum NOON state, we measure a visibility of 86 +/- 1%. The splitting ratio* (η) *for this device is 0.66 which results in an ideal visibility of 2*sqrt(η*(1- η)) which is about 94.7%. If we take into account the source visibility of 92%, the estimated visibility is 87% which is within the error of our measurement. There is no significant degradation of the quantum visibility due to additional spatial modes in the NOON state interferometer, and this degradation comes solely from the deviation of the splitting ratio from 0.5*.

*Reviewer #2 (Remarks to the Author):*

*1. I am happy with the passive mode conversion schemes pointed out by the authors, though as far as I know there is no experimental demonstration of such an arbitrary mode converter yet. In principle, a careful design and engineering of the device probably can achieve this challenging task. The costs probably are the fabrication of the nano-structures and computational complexity of design and optimization.*

We acknowledge the importance of this point and appreciate the constructive comments. As both nanoscale technology and computational design both advance, many of these issues can be addressed, and there are already works in the field that show this direction.[1–5]

*2. The discussion of active mode-MZI here is focusing on how to exploit high TO-materials and engineer the structure to reduce the power consumption of the mode-MZI. However, using these technical improvements, I am suspicious that the power consumption for a standard MZI can be reduced as well. Thus, the authors should compare this mode MZI with the normal MZI within the same scope.*

*As the authors state in the main text that"… phase shift between the fundamental and higher order modes as the heater is tuned…". When increasing the temperature, the refractive indices of both modes increase, partially cancelling the phase difference between them. This could be the reason that relative high power consumption is necessary to induce the phase difference between different modes. Also, this mode MZI would be difficult to implement in high dimensional case.*

We agree the mode-selective phase shift is a remaining challenge that requires further research. We have included a discussion in the supplementary with numerical simulations to show how the relative phase shift between the spatial modes is affected by various design parameters (see Supplementary Note 3 and Supplementary Fig. 2-3). Indeed, the power required to achieve a pi phase shift between two single mode waveguides is 7 times lower than for two spatial modes

within the multimode waveguide. As the reviewer suspects, this is because the heat delivered affects both modes to some extent and reduces the relative phase difference. We have shown that our use of a localized heater has improved this relative phase difference. To further address this issue, we show that in principle air trenches around the heater can create a sharper thermal gradient that reduces the power consumption by about 25%. With this same trench configuration for the single mode waveguide case, we see no improvement, so we did not include this in the Supplementary (see Fig. 1 below). We include a line in the main text to acknowledge this issue as follows (see lines 154-157): "The relatively high powers required to achieve a differential phase shift between the higher order modes requires further optimization. Simulations and extended discussion on this point are included in Supplementary Note 3 and Supplementary Fig. 2-3." Our analysis is limited to thermo-optic phase tuning. However, other electro-optic effects could be more localized. Further research in this area should include intermediate structures that utilize the inherently different coupling coefficients to apply larger differential phase shifts between the spatial modes. However, we would like to reiterate this is the first demonstration of an MZI using spatial waveguide modes and there is plenty of room for optimization.

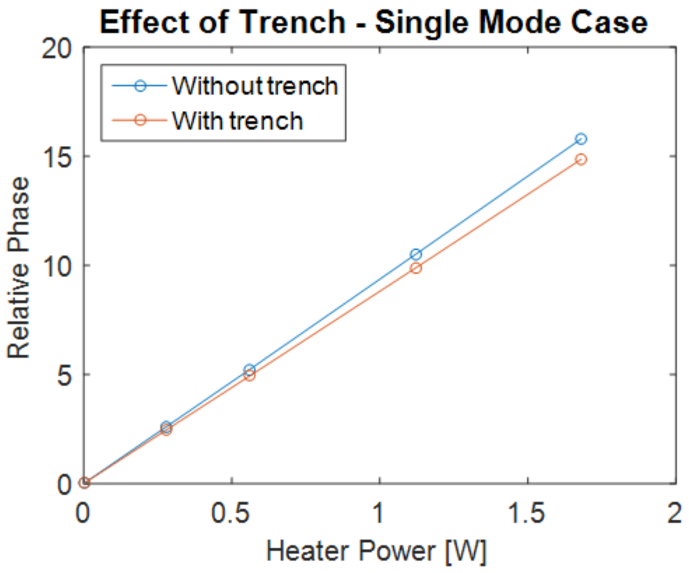


*Figure 1 Effect of trench on thermo-optic phase shift for single mode waveguide phase shifters. We use the same configuration as the multimode case that is shown in Supplementary Fig. 2c.*

*3. The motivation of this work is to move to higher dimensional quantum systems by the use of many orthogonal waveguide modes, though this work is a*

*demonstration of two modes case, it will be necessary to include a detailed discussion on how to scale it up using this technology.*

We agree this is very important, and in addition to our discussion section in the main text, we include an extended discussion of how this technology can be scaled up to operate on a larger number of orthogonal waveguide modes in the supplementary (see Supplementary Note 4 and Supplementary Fig. 4-5). Using the higher order waveguide modes in addition to other degrees of freedom will create a highly scalable system. We have included the following in the main text to highlight this detailed discussion in the supplementary (see lines 170-172): "We include an example of designing a three mode splitter and an extended discussion on the footprint and scalability of this platform in Supplementary Note 4 and Supplementary Fig. 4-5."

In the classical regime, mode-multiplexing is being used in addition to wavelength-multiplexing to scale up data transmission rates of both fiber and integrated communications systems without the need for additional lasers. Data transmission rates of 90 Gbps have been demonstrated using 3 spatial and up to 8 spatial modes have been multiplexed into a single waveguide. With 5 modes, current WDM systems could reach nearly 4.5 Tbps for a single multimode waveguide. This combined with the work towards nanostructuring for mode conversion shows significant potential for mode-encoding in the quantum regime.

*4. The authors have compared the directional path-BS with the new mode-BS in term of device footprint. It will be even more convincing to compare this mode-BS directly with the multimode interferometer path-BS, which are both naturally utilizing the multimodes of waveguide.*

As part of the discussion on scalability in Supplementary Note 4, we have included an estimate of the footprint using the multimode interferometer (MMI) path-BS and s-bends which reduce the path-encoded 2x2 beamsplitter footprint to 1000 um^2. We include a plot (see Supplementary Fig. 5) comparing the footprints of the path-encoded and waveguide-mode-encoded systems for NxN systems using 2x2 transformations. Here we see that the footprint of the mode-encoded device continues to scale more favorably with added modes. We believe this is a fair comparison of the two platforms because this is the most straightforward, reconfigurable, and simple implementation of arbitrary mode transformations.

Although, in theory, an NxN MMI can be constructed, it is important to note that experimentally there are very few demonstrations of greater than 4x4 MMI's because they are difficult to keep balanced with low insertion loss which we explain in the discussion. The splitter for N=4 using an MMI is about 4 times larger than the mode-encoded structure using 2x2 couplings, and this ratio grows slowly with increasing number of modes. However, the MMI is primarily limited to symmetric splitting.

The contribution of our work is precisely to show that the modes can be individually manipulated for full control over the transformation. In fact, to create variable ratio splitters in MMI's, it has been suggested to use multiplexed gratings within the mmi section. Therefore, the techniques we suggest here can be leveraged to improve path-encoded MMI designs as well.

*5. I do agree that path-encode and mode-encode would/should not be exclusively competing with each other. Indeed, the use of mode-encoding here could add more functionality for chip-scale quantum photonics. Together with the recent demonstration of polarization, mode, path encoding conversion in L.-T. Feng, et. al. Nat Commun 7, 11985 (2016) and other related works, it is exhibiting the promise future of potential quantum information applications.*

--

*Work in a similar vein of that recently been published in Nature Communications, where Feng et. al. reported an "On-chip coherent conversion of photonic quantum entanglement between different degrees of freedom", and used them to demonstrate the two-photon two-mode HOM and entanglement transfer and manipulation.*

*To achieve the mode-BS, Feng et. al. used a polarization-mode conversion scheme, while here this work exploited a new grating structure as the mode-BS. Feng et al acknowledge in their paper that "while this report was being written, quantum interference between transverse waveguide modes was realized" – referencing this work. It would appear the paper under review has taken much longer to get to press than the Feng paper, although it was perhaps performed at a very similar time. So, although the work is somewhat similar in goal, this is an important topic and I would suggest that it should be published in Nat Communications, providing*

*a nice contrast to the work of Feng..*

*Ideally I would have liked for the authors to realize higher mode demonstration to verify their claims of scalability and show its advantage of using their mode-BS and mode-multiplexer for useful high dimensional quantum information systems.*

We agree with the reviewer that this work is a good contrast to the work of Feng et al. It is important to note that these other degrees of freedom can be combined with mode-encoding. We have cited this work in our original submission (see lines 37-39). Mode-encoding offers a powerful new ingredient in this already impressive and promising toolbox for integrated quantum optics. While they show the conversion between polarization, path, and spatial mode interference, we focus on showing the utility of the spatial mode degree of freedom which has the capacity to scale these integrated quantum systems dramatically. We show beam-splitting and phase shifting for quantum HOM and NOON interference entirely in the spatial mode degree of freedom. To support our claims of scalability of the waveguide mode platform, we include a detailed discussion as noted in the previous point (see Supplementary Note 4).

*Reviewer #4 (Remarks to the Author):*

*The manuscript details the demonstration of optical information processing on a chip making use of spatial modes and has already been through a round of review. The results are well presented and clear. Technical questions raised in the previous round of refereeing have been satisfactorily addressed by authors. Since the remaining questions seem to be over impact and novelty I'll focus on this aspect.*

*Pretty much every conceivable degree of freedom of the optical field has been exploited for photonic quantum information processing in free space optics. The most common are path and polarisation, but also angular momentum, spatial mode, frequency, temporal time-binning, and various combinations have been used. Some degrees of freedom are currently easier to manipulate with high precision than others but the driving force to explore others is the need to drastically scale up optical QIP and any advantage in doing so may prove useful.*

*It is also clear that, other than for quantum communication, integrated optical devices are the path forward. To date there is comparatively little exploration of alternative degrees of freedom of the light field in integrated devices so the manuscript is immediately novel in this sense. The fact that the interference experiments demonstrated (HOM dip and two-photon NOON state) are not novel and not of record breaking fidelity is not the important point, the important point is that the transverse spatial modes can be manipulated with high fidelity and relatively easily. This is a first-in-class demonstration of such techniques.*

*More than this, the achieved fidelities are respectable. They are certainly not the highest, especially in comparison with free space, but then those technologies have benefitted from decades of technical development. The state of the art is barely more than a handful of photons and to do something truely useful may require hundreds if not thousands of photonic qubits so it is much too early to rule out any particular approach. The importance of the demonstrated experiment is that it shows the same lithography technology used in creating path encoded devices can also give access to spatial mode encoding. If this is to be the eventual preferred approach to photonic QIP can't be said but it brings much needed diversification of approaches. Comparisons with other specific experiments that where raised by the previous referees have been addressed satisfactorily.*

*My recommendation is to accept the manuscript for publication.*

We thank the reviewer for his/her favorable comments and for highlighting the importance of our work in regards to its novelty. As the reviewer pointed out, we believe that mode encoding can be used in tandem with other degrees of freedom of the light field to scale up optical QIP, and our demonstration on an integrated platform is a significant demonstration in this regard.

References:


1. Lu, J. & Vuckovic, J. Objective-first design of high-efficiency, small-footprint couplers between arbitrary nanophotonic waveguide modes. *Opt. Express* **20,** 7221–7236 (2012).
2. Gabrielli, L. H., Liu, D., Johnson, S. G. & Lipson, M. On-chip transformation optics for multimode waveguide bends. *Nat. Commun.* **3,** 1217 (2012).
3. Ohana, D. & Levy, U. Mode conversion based on dielectric metamaterial in silicon. *Opt. Express* **22,** 27617–27631 (2014).
4. Piggott, A. Y. *et al.* Inverse design and demonstration of a compact and broadband on-chip wavelength demultiplexer. *Nat. Photonics* **9,** 374–377 (2015).
5. Shen, B., Wang, P., Polson, R. & Menon, R. An integrated-nanophotonics polarization beamsplitter with 2.4 × 2.4 μm2 footprint. *Nat. Photonics* **9,** 378–382 (2015).

REVIEWERS' COMMENTS:

Reviewer #2 (Remarks to the Author):

Considering the authors' revisions and responses, I am happy with the publication of this work in Nat. Commun.
